# Siglec-7 represents a glyco-immune checkpoint for non-exhausted effector memory CD8+ T cells with high functional and metabolic capacities

**DOI:** 10.3389/fimmu.2022.996746

**Published:** 2022-09-23

**Authors:** Quentin Haas, Nikita Markov, Lukas Muerner, Viviana Rubino, Andrej Benjak, Monika Haubitz, Gabriela M. Baerlocher, Charlotte K. Y. Ng, Christian Münz, Carsten Riether, Adrian F. Ochsenbein, Hans-Uwe Simon, Stephan von Gunten

**Affiliations:** ^1^ Institute of Pharmacology, University of Bern, Bern, Switzerland; ^2^ Graduate School for Cellular and Biomedical Sciences, University of Bern, Bern, Switzerland; ^3^ Bern Center for Precision Medicine (BCPM), University of Bern, Bern, Switzerland; ^4^ Department of Medical Oncology, Inselspital, Bern University Hospital, University of Bern, Bern, Switzerland; ^5^ Department for BioMedical Research (DBMR), University of Bern, Bern, Switzerland; ^6^ Experimental Hematology, Department for BioMedical Research, University of Bern, Bern, Switzerland; ^7^ Viral Immunobiology, Institute of Experimental Immunology, University of Zurich, Zurich, Switzerland; ^8^ Department of Clinical Immunology and Allergology, Sechenov University, Moscow, Russia; ^9^ Laboratory of Molecular Immunology, Institute of Fundamental Medicine and Biology, Kazan Federal University, Kazan, Russia; ^10^ Institute of Biochemistry, Brandenburg Medical School, Neuruppin, Germany

**Keywords:** Siglec-7, CD8+ T cells, acute myeloid leukemia, immune checkpoint, tumor immunity and immunotherapy, sialoglycans, hypersialylation

## Abstract

While inhibitory Siglec receptors are known to regulate myeloid cells, less is known about their expression and function in lymphocytes subsets. Here we identified Siglec-7 as a glyco-immune checkpoint expressed on non-exhausted effector memory CD8+ T cells that exhibit high functional and metabolic capacities. Seahorse analysis revealed higher basal respiration and glycolysis levels of Siglec-7^+^ CD8+ T cells in steady state, and particularly upon activation. Siglec-7 polarization into the T cell immune synapse was dependent on sialoglycan interactions *in trans* and prevented actin polarization and effective T cell responses. Siglec-7 ligands were found to be expressed on both leukemic stem cells and acute myeloid leukemia (AML) cells suggesting the occurrence of glyco-immune checkpoints for Siglec-7^+^ CD8+ T cells, which were found in patients’ peripheral blood and bone marrow. Our findings project Siglec-7 as a glyco-immune checkpoint and therapeutic target for T cell-driven disorders and cancer.

## Introduction

Siglecs are surface receptors that are differentially and broadly expressed on immune cells, and have recently emerged as critical immune checkpoints in health, inflammatory disease and cancer (1–4). These lectin receptors are thought to protect from autoreactivity by recognition of sialic-acid containg carbohydrates (sialoglycans) as so-called self-associated molecular patterns (SAMPs) (5). However, such sialoglycan ligands are overexpressed in a variety of different types of malignancies (6), eventually leading to tumor immune evasion by engagment of inhibitory Siglec receptors (7). Whereas Siglec-mediated suppression of immune responses could be therapeutically exploited in autoimmune and inflammatory disease, targeting Siglecs might restore anti-tumor responses as a form of normalization cancer immunotherapy (8). Indeed, a broad range of anti-Siglec therapies are currently explored in pre-clinical studies and first candidate drugs have been forwarded to evaluation in clinical trials (1, 9, 10).

The expression of Siglecs has been reported to be low or absent on human T cells (11–13). However, we and others recently reported enhanced Siglec expression on peripheral blood and tumor infiltrating T cells of cancer patients (14, 15). Notably, in melanoma tissues the majority of tumor-infiltrating lymphocytes consisted of Siglec-9^+^ CD8^+^ T cells (15). Mechanistic studies revealed that Siglec-7 and -9 can directly suppress TCR signaling (12, 15), which results in synergistic effects yet involves disparate signaling pathways compared to the immune checkpoint receptor PD1 (15). Together, these observations invigorate the interest in the role of Siglecs for T cell biology (3).

In the present study, we identified Siglec-7 as a glyco-immune checkpoint receptor on non-exhausted effector memory CD8^+^ T cells with high functional and metabolic capacities, and a history of previous clonal expansion. Notably, Siglec-7 was found to congregate within the T cell immune synapse which was associated with reduced CD8^+^ T cell effector functions including cytotoxicity and cytokine production. Primary leukemic cells and stem cells from patients with acute myeloid leukemia (AML), a malignancy so far ineffectively treated with immune checkpoint therapy, expressed high levels of Siglec-7 ligands. This might adversily affect effector responses of Siglec-7^+^ CD8^+^ T cells, which were found in the blood and bone marrow of AML patients. The understanding of Siglec-7 as a clonality-associated glyco-immune checkpoint might inspire novel therapeutic approaches to T cell-associated disorders and cancer.

## Materials and methods

### Cells and tissues

Blood from healthy donors was collected upon informed consent or buffy coats were purchased from the Blood Transfusion Center of Bern, Switzerland. Mononuclear cells were obtained by density centrifugation using Pancoll solution (PAN-Biotech, Aidenbach, Germany). For functional experiments, CD8+ T-cells were isolated using the EasySep™ Human CD8^+^ T Cell Isolation Kit (StemCell Technologies, Vancouver, Canada), according to the manufacturer’s instructions. The purity of isolated cells was >95%. For experiments with CD8^+^ T cells subsets, cells were isolated using fluorescence-activated cell sorting (FACS Aria, BD Biosciences, Franklin Lakes, USA). Informed consent was obtained from all patients prior to tissue sample collection. Peripheral blood and BM aspiration samples were obtained from untreated AML patients at the University Hospital of Bern (Switzerland) after informed consent. Samples were stored in liquid nitrogen. AML bone marrow samples were thawed using citrate-dextrose solution in a concentration 1:10 in order to avoid cell clumping. Samples were washed and resuspended in FCS-containing medium prior to staining. All studies using human material were in accordance with the Helsinki Declaration and approved by the cantonal ethics committee of Bern, Switzerland. Written informed consent was received from participants prior to inclusion in the study. For redirected cytotoxic assay, the mouse mastocytoma cell line P815 (American Type Culture Collection ATCC, Manassas, VA, USA) was used exclusively between passage 5 and 8. No mycoplasma testing was performed.

### Cell culture

Isolated CD8^+^ T cells were cultured in RPMI medium (Sigma-Aldrich, Missouri, USA) containing 10% fetal calf serum (FCS) (Life Technologies, Waltham, USA) and 1% penicillin/streptomycin (Life Technologies) supplemented or not with 100 U/mL rhIL-2 (Peprotech, USA). When required, cells were activated with plate-bound αCD3 (1 µg/mL; OKT-3, BioXcell, Lebanon, USA) and soluble αCD28 (1 µg/mL, BioLegend, San Diego, USA) antibodies for 1 h at 37°C in supplemented medium. The mouse mastocytoma cell line P815 was cultured in DMEM medium (Sigma-Aldrich) containing 10% fetal calf serum (FCS) (Life Technologies) and 1% penicillin/streptomycin (Life Technologies).

### Monoclonal antibodies and cell labeling

PBMCs, lymphocytes isolated from tissues or purified CD8^+^ T cells, were labeled using fluorescent mAbs directed against surface molecules (20 min at 4°C), washed in PBS with 0.2% BSA (Sigma-Aldrich), and acquired using FACSVerse or FACSLyric (BD Biosciences, Franklin Lakes, NJ, USA). When required, cells were blocked using FC-block (human Trustain FcX, BioLegend, San Diego, CA, USA), and viability was analyzed using the Zombie NIR or Violet viability kit (BioLegend). Cells were labeled either directly *ex vivo* or, where indicated, after 30 min of treatment with 25 mU neuraminidase (Roche Diagnostics, Rotkreuz, Switzerland) at 37°C. All mAbs were purchased from BioLegend, with the exception of fluorochrome-conjugated antibodies against CD3, CD8, CLA, CD45RA, LAG3, CXCR3, CCR4, and CCR7 (BD Bioscience); Siglec-9, CCR1 and CCR7 (R&D Systems, Minneapolis, USA); Siglec-7 (Beckman Coulter, Brea, CA, USA), and TNF-α (eBioscience, Waltham, MA, USA). Each mAb was titrated on PBMCs before use.

### RNA analysis on TCGA database

TCGA RNA-seq data were retrieved from the GDC portal (https://portal.gdc.cancer.gov/) using the following filtering rules: Disease Type IS myeloid leukemias AND Workflow Type IS HTSeq - Counts AND Experimental Strategy IS RNA-Seq. The dataset consisted of 818 samples derived from 740 patients (274 primary cancer/bone marrow; 265 primary cancer/peripheral blood; 140 recurrent cancer/bone marrow; 139 recurrent cancer/peripheral blood). We defined the top/bottom 25% (n = 205) samples based on *SIGLEC7* (ENSG00000168995.12) and *SIGLEC9* (ENSG00000129450.7) expression values (transcript per million), respectively. Differential gene expression analysis was done between the “top” and “bottom” groups of samples (separately for Siglec-7 and Siglec-9) using DESeq2 and raw gene counts. Gene set enrichment analysis was done with GSEA 4.0.3 and the MSigDB v7.0 (16), using the “hallmark gene sets”, and the following options: -nperm 1000 -scoring_scheme weighted -plot_top_x 200 -rnd_seed timestamp -set_max 500 -set_min 15. Input genes were all the differentially expressed genes with a padj value below 0.05. Plots were made in R 3.6.3 using ggplot2.

### Telomere length measurement by automated multicolor flow-FISH

For telomere length analysis, human CD8^+^ T cells Siglec-9^+^, Siglec-7^+^ and Siglec-9^-^/7^-^ subsets were isolated from the peripheral blood of 3 healthy donors by fluorescence-activated cell sorting as described above. Telomere length measurement by *in situ* hybridization and flow cytometry (automated multicolor flow-FISH) was performed as previously done (6): Briefly, 2.5 × 10^3^ to 2 × 10^6^ cells were used for *in situ* hybridization. Cells were incubated with 170 μL hybridization mixture containing 75% deionized formamide (Sigma-Aldrich), 20 mM Tris (pH 7.1; Sigma-Aldrich), and 1% BSA (Sigma-Aldrich) with no probe (unstained) or 0.3 μg/mL telomere-specific FITC conjugated (C_3_TA_2_)_3_ peptide nucleic acid (PNA) (Applied Biosystems, Foster City, USA). Denaturation was done at 87°C for 15 min, and hybridization was performed in the dark and at room temperature (RT) for 90 min. Excess and nonspecifically bound telomere PNA probes were removed by 4 washing steps at RT using 1 mL washing solution containing 75% formamide, 10 mM Tris, 0.1% BSA, and 0.1% Tween 20 (Sigma-Aldrich), followed by 1 × 1 mL wash with a solution containing PBS, 0.1% BSA, and 0.1% Tween 20 at RT. DNA counterstaining was performed using a solution containing Sheath Fluid (BD Bioscience), 0.1% BSA, and a subsaturating amount of LDS 751 (0.01 μg/mL; Invitrogen, Waltham, MA, USA) overnight. Acquisition of telomere fluorescence was performed using FACSCalibur (BD Biosciences). For each sample, unstained and telomere-stained samples were tested. FlowJo version 10 (Tree Star Inc.) was used for analysis of telomere length in the specific cell subsets. Specific telomere fluorescence was determined as the difference between the fluorescence of the stained samples minus the (auto-) fluorescence of the corresponding unstained sample. Using calibration beads and an internal standard of cow thymocytes, the telomere fluorescence was calculated into kilobases of telomere length.

### Intracellular cytokine measurements

Isolated CD8^+^ T cells were stimulated for 1 h at 37°C in 5% CO_2_ with αCD3 (1 µg/mL, plate bound) and αCD28 (1 µg/mL, soluble) antibodies or with αCD3 mAb-coated P815 cells (see below). Thereafter, GolgiPlug and GolgiStop (BD Biosciences) were added to the cultures followed by incubation for 5 h. Cells were spun down and incubated with fluorochrome-conjugated mAbs for multiparametric flow cytometric analysis, when required. Cells were then washed, fixed with 2% paraformaldehyde in PBS, permeabilized, and stained intracellularly with fluorochrome-conjugated mAbs against cytokines. Finally, cells were washed and analyzed on a BD FACSVerse (BD Biosciences). Data were analyzed with FlowJo 10.0.6 software (Tree Star Inc., Ashland OR, USA).

### Redirected cytotoxicity assay

Cytolytic CD8^+^ T cell activity was evaluated in a redirected cytotoxicity assay against P815 cells. To this end, the P815 cells were coated with 20 μg/mL of αCD3 (OKT-3) for 1 h. When indicated, P815 cells were treated with neuraminidase (25 mU, Roche Diagnostics) for 30 min at 37°C. CD3-coated P815 cells were co-cultured (3:1 E/T ratio) with CD8^+^ T cells at 37°C. After 4 h of incubation, the specific lysis of P815 cells was assessed by measuring the LDH activity in the supernatant using the Cytotoxicity Detection Kit^PLUS^ LDH (Roche), according to the manufacturer’s instructions. Specific lysis was calculated as (experimental – spontaneous release)/(total – spontaneous release)*100 and expressed as a fold change between treated and untreated groups (specific lysis fold change).

### Transwell cell migration assay

2 x 10^5^ Siglec-7^+^ and Siglec-7^-^ CD8^+^ T cells were resuspended in 200 µL RPMI medium and preactivated using αCD3 and αCD28 co-stimulation for 1 h as described above. Cells were loaded on Transwell inserts with 5 μm pores (Stemcell). Transwell inserts were placed over 24 wells plate well containing 600 μL serum-free RPMI, supplemented with or without 100 ng/mL of rhRANTES (CCL5, BioLegend) or rhCXCL9 (BioLegend). Cells were incubated for 4 h at 37°C and then analyzed using flow cytometry (see above).

### TCRVβ sequencing

CD8^+^ T cells were isolated from peripheral blood of healthy donors and separated by fluorescence-activated cell sorting into Siglec-9^+^, Siglec-7^+^ and Siglec-9^-^/7^-^ populations as previously described. Genomic DNA from CD8^+^ T cells subsets was extracted using NucleoSpin^®^ Tissue kit from Macherey-Nagel according to the manufacturer instructions. Genomic DNA quantity and purity were assessed through spectrophotometric analysis. 1.47 to 29.1 ng/µL of genomic DNA were analyzed by high-throughput sequencing of the TCRVβ using the ImmunoSEQ immune profiling platform at the survey level (Adaptive Biotechnologies Corp, Seattle, WA), which represents a detection capacity of 1 cell in 40’000. Raw data can be retrieved from the immuneACCESS repository (DOI:10.21417/haas-2022-fi URL: https://clients.adaptivebiotech.com/pub/haas-2022-fi).

### Flow cytometric quantification of Siglec ligands on AML cells

Detection of Siglec-7 ligands by flow cytometry was performed as previously described (6). AML-derived samples were analyzed as previously described (17). In brief, non-specific antibody binding by Fc receptors was blocked using 100 μL of Fc Receptor Blocker (Innovex Biosciences, Richmond, CA, USA) and dead cells were excluded from analysis by staining with a Fixable Viability Dye (ThermoFisher). For the Siglec ligands staining, recombinant human Siglec-7-hFc (R&D Systems, Minneapolis, MN, USA) was pre-incubated with PE-conjugated goat anti-human Ig (Jackson ImmunoResearch Laboratories, West Grove, USA) for 1 h at 4°C and then applied to the samples for 1 h at RT together with lineage-associated antibodies. Lineage-positive cells were stained with biotinylated αCD2, αCD14, αCD16, αCD19, αCD56, and αCD235, together with fluorophore-conjugated αCD8, αCD4, αCD45, αCD34, αCD38 (all from BioLegend), followed by a second step with streptavidin-FITC conjugate (BD Biosciences). Cells were washed and analyzed on a BD FACSVerse or BD FACSlyric (BD Biosciences). Data were analyzed using the FlowJo 10.0.6 software (Tree Star Inc.).

### Immune synapse analysis

Isolated CD8^+^ T cells subgroups were obtained by cell sorting as previously described. P815 cells were incubated with 20 μg/mL of αCD3 (OKT-3) for 1 h. When indicated, P815 cells were treated with neuraminidase (25 mU, Roche Diagnostics). CD3-coated P815 cells were co-cultured (3:1 E/T ratio) with CD8^+^ T cells subgroups for 30 min. After incubation, the cells were centrifuged on poly-lysine (Sigma)-treated coverslips, fixed using 3% paraformaldehyde for 20 min at 4°C and permeabilized with Triton X-100 for 1 min. After extensive washing with fish skin gelatin buffer (PSG), αSiglec-7 antibody and BODIPY 488 Phalloidin (actin dye, Life Technologies) were applied for 1 h at RT. After another round of washing, the secondary antibody (goat anti-mouse, Alexa-Fluor 555, Invitrogen) was applied for 1 h at RT. Coverslips were mounted on slides (Fisherbrand, Thermo Fisher) using ProLong Gold anti-fade reagent (Invitrogen). The mounted slides were cured for 2 d in the dark at RT. Long-time storage at 4°C. Conjugates were analyzed by confocal laser scanning microscopy (LSM510, Carl Zeiss, Jena, Germany). Acquired images were analyzed using the ImageJ software version 1.51 (NIH, Bethesda, MD, USA).

### Analysis of bioenergetic profiles

CD8^+^ T cells subsets were isolated from healthy donors as previously described. T cells were either analyzed directly following sorting or were activated for 7 d using αCD3 and αCD28 (each at 1 µg/mL) as described above. Bioenergetic profiles were assesed using a seahorse approach (18). Briefly, to overcome machine detection limitations, sorted CD8^+^ T cells from 2 to 4 patients were pooled together for each condition. XF96 cell culture microplate (Agilent, Santa Clara, USA) was treated with 30 μL poly-D-lysine (Sigma-Aldrich) for 1–2 h, before washing with ddH_2_O. Sorted CD8^+^ T cells subsets were harvested, washed in non-buffered DMEM containing 25 mM glucose, 2 mM L-glutamine and 1 mM sodium pyruvate (Seahorse XF DMEM medium, Agilent). Cells were then resuspended in medium at a concentration of at least 5×10^6^ cells/mL. 40 μL of cells were plated into the bottom of the analysis wells (=2x10^5^ cells/well). Cells were centrifuged 5 min at 400xg to adhere and form a monolayer at the bottom of the plate. 140 μL of medium was added to each well and cells were incubated at 37°C in a non-CO_2_ incubator for 60 min. 10x stocks of compounds (oligomycin, fluoro-carbonyl cyanide phenylhydrazone, rotenone, antimycin A and 2-deoxy-D-glucose; all purchased from Sigma-Aldrich) in medium were prepared and loaded into delivery ports of XFe96 sensor cartridges. Oxygen consumption rate (OCR) and extracellular acidification rate (ECAR) were measured under basal conditions and in response to sequentially injected compounds at a final concentration of 1 μM oligomycin, 1.5 μM fluoro-carbonyl cyanide phenylhydrazone and 100 nM rotenone + 1 μM antimycin A and 50 mM of 2-deoxy-D-glucose using the XF-96 Extracellular Flux Analyzer (Agilent).

### Statistics

Statistical analysis was performed using Prism 7.0 (GraphPad Software, San Diego, CA, USA). For quantitative comparisons between two groups the paired Student’s *t* test and between multiple groups one-way ANOVA tests with Bonferroni or Dunn posttest were used. All statistical tests were two-sided and *P* < 0.05 was considered significant. Unless otherwise indicated, data represent mean ± standard deviation (SD).

## Results

### Siglec-7 defines a distinct subset of effector memory CD8^+^ T cells

**Figure 1:**
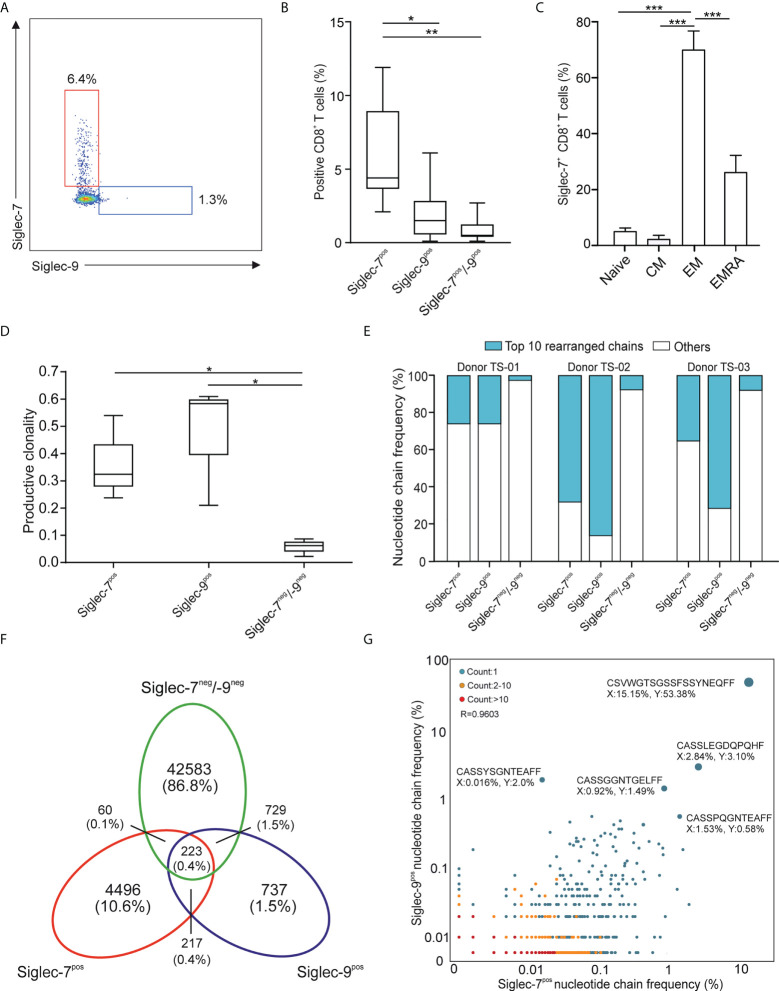
Siglec-7 defines a unique subset of effector memory CD8^+^ T cells in healthy donor peripheral blood. **(A, B)** Representative flow cytometry plot **(A)** and quantitative analysis (n=23) **(B)** for comparison of Siglec-7 and Siglec-9 surface expression on healthy donor peripheral blood CD8^+^ T cells. **(C)** Composition of Siglec-7^+^ CD8^+^ T cell subsets, including naive, central memory (CM), effector memory (EM), and CD45RA^+^ effector memory (EMRA) cells (n=8). **(D–G)** TCRvβ chain analysis of peripheral blood CD8^+^ T cell subsets. Productive clonality **(D)** and clonotype frequency distribution **(E)** for three individual donors. Venn diagram **(F)** and scatter plot **(G)** representation displaying clonotypes distribution among CD8^+^ T cell subsets for donor TS-02. Statistical analysis was performed by one-way ANOVA followed by **(B, C)** Bonferroni or **(D)** Dunn posttest. *P < 0.05; **P < 0.01; ***P < 0.001. Error bars, SD.

In line with earlier reports (11, 12), we observed a subset of Siglec-7^+^ CD8^+^ T cells in the peripheral blood of healthy individuals (**Figures 1A, B** and **Supplementary Figure 1A**). Our flow cytometric analysis revealed that these cells represented a distinct and more frequent subtype compared to Siglec-9^+^ CD8^+^ T cells, and were predominantly negative for NK cell (CD56) and natural killer T cell (NKT, TCR Vα24-Jα18) markers (**Supplementary Figures 1B, C**). Unmasking of potential sialoglycan ligands bound *in cis* by neuraminidase had no further effect on the flow cytometric assessment of Siglec-7 on CD8^+^ T cells (**Supplementary Figure 2**). Further phenotypic analysis based on CCR7 and CD45RA cell surface expression (19), indicated that Siglec-7^+^ CD8^+^ T cells predominantly constitute effector memory (EM), and to a lesser extent effector memory cells re-expressing CD45RA (EMRA), T cell subsets (**Figure 1C**).

Deep sequencing of TCRVβ chains on genomic DNA performed by multiplex polymerase chain reaction (PCR) assays (20), was performed to decipher the TCR repertoires of Siglec-7^+^, Siglec-9^+^ and Siglec-7/9^-/-^ CD8^+^ T cell subsets from three donors (TS-01, TS-02, and TS-03). The low clonality scores of Siglec-7/9^-/-^ CD8^+^ T was consistent with the highly diverse repertoire of circulating T cells (**Figure 1D**). In contrast, both Siglec-7^+^ and Siglec-9^+^ CD8^+^ T cells exhibited high normalized productive clonality scores based on diversity and sample entropy, indicative of limited TCR rearrangements and enriched clones within these subsets. Corroborating results were obtained when maximum productive frequency was assessed (**Supplementary Figure 3**). Furthermore, a frequency distribution analysis of clonotypes based on the 10 most prevalent nucleotide TCRvβ chains confirmed the clonal expansion pattern of Siglec-7^+^ and Siglec-9^+^ CD8^+^ T cells (**Figure 1E**).

We went on to compare the clonotype repertoires of Siglec-7^+^, Siglec-9^+^ and Siglec-7/9^-/-^ subsets of peripheral blood T cells (**Figure 1F** and **Supplementary Figure 4**). The Siglec-7^+^ T cell subset enclosed a higher proportion (2.7-10.6%) of the total of all clonotypes, as compared to the Siglec-9^+^ subset (0.9-2.8%) (**Figure 1F** and **Supplementary Figure 4**), eventually in line with their higher occurrence in the circulation. The analysis of the TCRVβ nucleotide chain distribution and clonotypes frequency in Siglec-7^+^ CD8^+^ T cells (x-axis) and Siglec-9^+^ CD8^+^ T cells (y-axis) from donor TS-02 confirmed the distinct clonotype profile of the Siglec-7^+^ subset (**Figure 1G**). Taken together, these data suggest that Siglec-7^+^ CD8^+^ T cells, similarily to their Siglec-9 positive counterparts, are expanded oligoclonal effector memory T cells, but represent a distinct subset in light of the TCR repertoire.

### High metabolic capacities of Siglec-7^+^ CD8^+^ T cells in steady state and upon activation

**Figure 2:**
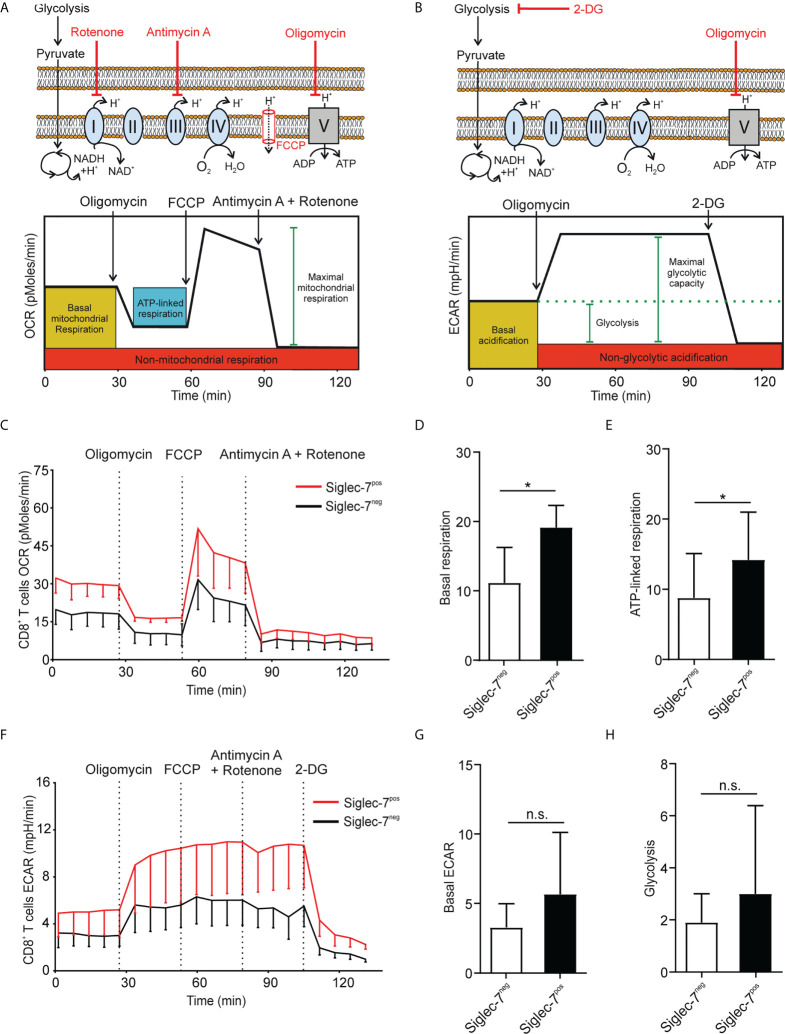
Siglec-7+ CD8+ T cells display a higher oxidative phosphorylation capacity at steady state. **(A, B)** Schematic illustrations of expected effects of oligomycin (ATPase inhibitor), FCCP (mobile ion carrier), 2-deoxy-D-glucose (2-DG, glucoprivic agent), and antimycin A (cytochrome C reductase blocker) and rotenone (complex I blocker) on oxygen consumption rate (OCR) **(A)** and extracellular acidification rate (ECAR) **(B)**. **(C–H)** Comparative OCR or ECAR analysis of isolated Siglec-7^+^ and Siglec-7^-^ CD8^+^ T cells from healthy donors by Seahorse (three independent experiments with 2-4 donors each, 11 donors in total). OCR profile **(C)**, basal respiration (steady state mitochondrial respiration) **(D)**, and ATP-linked respiration (ΔOCR after oligomycin injection) **(E)**. ECAR profile **(F)**, basal acidification (ECAR at steady state) **(G)**, and glycolysis (ΔECAR at steady state minus non-glycolytic acidification in response to 2-DG injection) **(H)**. Statistical analysis was performed by paired *t* test **(D, E, G, H)**. *P < 0.05; n.s., not significant. Error bars, SD **(D, E, G, H)** or SEM **(C, F)**.

Metabolism plays a key role in immune cell functionality and activated effector T cells elevate aerobic glycolysis and oxidative phosphorylation (OXPHOS) (21, 22). Using Seahorse technology, we investigated the oxygen consumption rate (OCR) and extracellular acidification rate (ECAR) in presence of oligomycin (complex V blocker), FCCP (mitochondrial uncoupler), 2-deoxy-D-glucose (2-DG, glycolysis inhibitor), or combined antimycin A (complex III blocker) and rotenone (complex I blocker) treatment (**Figures 2A, B**). OCR analysis of sorted CD8^+^ T cell subsets revealed significantly higher basal respiration (**Figures 2C, D**) and ATP-linked respiration (**Figure 2E**) rates of Siglec-7^+^ CD8^+^ T cells at steady state. Compared to their Siglec-7 negative counterparts, Siglec-7^+^ CD8^+^ T cells also exhibited an overall amplified ECAR profile (**Figure 2F**), and a trend towards higher basal ECAR (**Figure 2G**) and glycolysis (**Figure 2H**). The increased OXPHOS capacity of Siglec-7^+^ CD8^+^ T cells at steady state matched the metabolic profile previously described for effector T cells (22–24), corresponding with the surface marker analysis.

**Figure 3:**
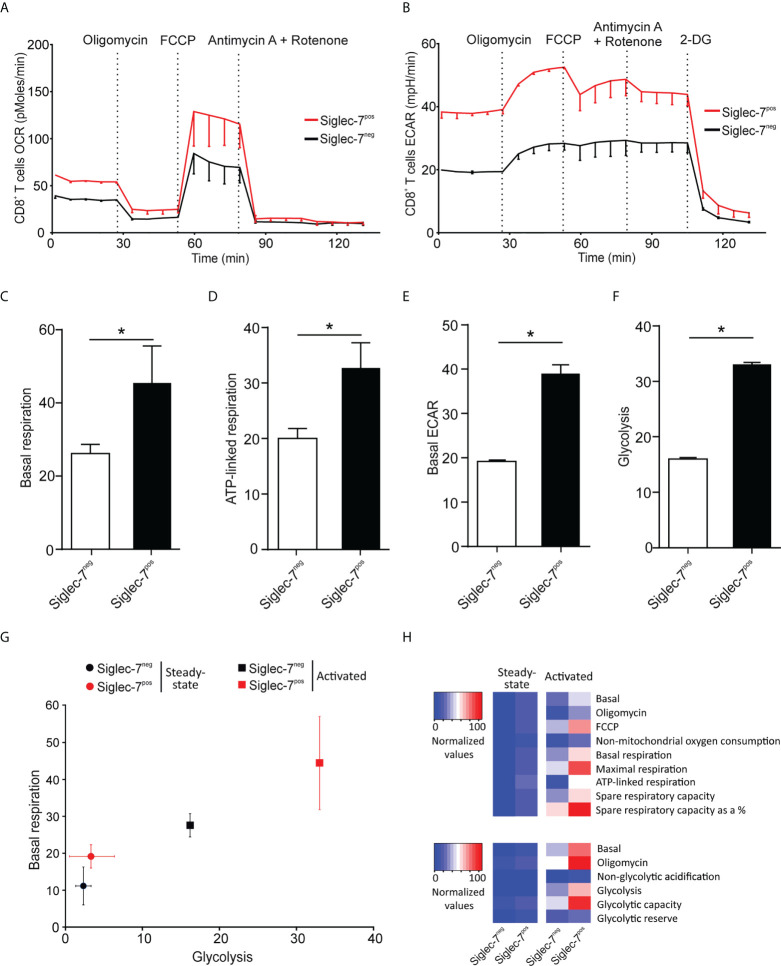
Upon activation Siglec-7^+^ CD8^+^ T cells exhibit a higher increase in oxidative phosphorylation and glycolysis compared to Siglec-7^-^ CD8^+^ T cells. Seahorse analysis of Siglec-7^+^ or Siglec-7^-^ CD8^+^ T cells after 7 days of co-stimulation by αCD3 and αCD28 (each at 1 μg/mL) (three independent experiments with 2-4 donors each, 11 donors in total). Oxygen consumption rate (OCR) **(A)** and extracellular acidification rate (ECAR) **(B)** profiles, basal respiration (steady state mitochondrial respiration) **(C)**, and ATP-linked respiration (ΔOCR after oligomycin injection) **(D)**, basal acidification (ECAR at steady state) **(E)**, and glycolysis (ΔECAR at steady state minus non-glycolytic acidification in response to 2-DG injection) **(F)**. **(G, H)** Comparison of the metabolic profiles of αCD3 and αCD28 co-stimulated and unstimulated Siglec-7^+^ and Siglec-7^-^ CD8^+^ T cells. Plot representation of basal respiration and glycolysis rates **(G)** and heatmap representation **(H)**. Statistical analyses were performed by paired *t* test **(C–F)**. *P < 0.05. Error bars, SEM **(A, B)** or SD **(C–G)**.

Next, we compared the metabolic behavior of Siglec-7^+^ and Siglec-7^-^ CD8^+^ T cells upon activation by αCD3 and αCD28 mAbs co-stimulation for a duration of 7 days (25). In analogy to steady state conditions, upon activation Siglec-7^+^ CD8^+^ T cells displayed elevated OCR and ECAR profiles (**Figures 3A, B**), significantly increased basal respiration (**Figure 3C**), and augmented ATP-linked respiration (**Figure 3D**). Furthermore, upon activation the basal ECAR (**Figure 3E**) and glycolytic capacity (**Figure 3F**) of Siglec-7^+^ CD8^+^ T cells raised dramatically compared to Siglec-7^-^ CD8^+^ T cells at steady-state. **Figure 3G** highlights the higher basal respiration and glycolysis levels of Siglec-7^+^ CD8^+^ T cells compared to Siglec-7^-^ CD8^+^ T cells in steady state, and particularly upon activation. The heatmap in **Figure 3H** summarizes the key metabolic characteristics, illustrating the bioenergetic advantage of activated Siglec-7^+^ CD8^+^ T cells over Siglec-7^-^ cells, particularly in regard of ATP-linked respiration and spare respiratory capacity.

### Siglec-7^+^ CD8^+^ T cells represent a non-exhausted and effective cell subset

**Figure 4:**
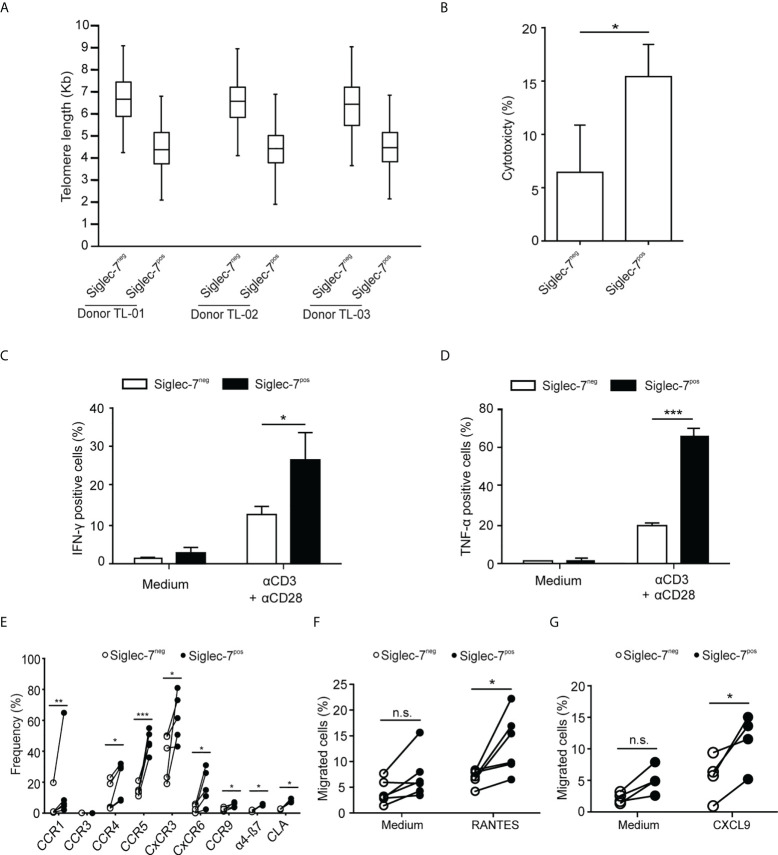
Siglec-7+ CD8+ T cells display a highly proliferative and functional phenotype. **(A)** Telomere length analysis of CD8^+^ T cell subsets from three healthy donors. Box plot representation indicating 25^th^ to 75^th^ percentiles with median; error bars, 1^st^ to 99^th^ percentiles. **(B)** Redirected cytotoxicity of CD8^+^ T cell subtypes upon co-culture with αCD3–loaded P815 tumor cells for 4 h (n=4). **(C, D)** Flow cytometric quantitative analysis of intracellular IFN-γ or TNF-α production by CD8^+^ T cell subsets following 5 h of culture upon costimulation by αCD3 and αCD28 (each at 1 μg/mL; n=5). **(E)** Flow cytometric quantitative analysis of chemokine receptors on the surface of CD8^+^ T cell subsets (n=5). **(F, G)** Migration of CD8^+^ T cell subsets towards 100 ng/mL of RANTES (E, n=6) or CXCL9 (F, n=5) through transwell inserts with 5 μm pores after 4 h incubation. Statistical analyses performed by paired *t* test **(C–F)** or one-way ANOVA followed by Bonferroni posttest **(A)**. *P < 0.05; **P < 0.01; ***P < 0.001. n.s., not significant. Error bars, SD.

We went on to further explore the phenotypic and functional characteristics of Siglec-7^+^ CD8^+^ T cells. Previously, we reported that Siglec-9^+^ T cells (15), but not Siglec-9^+^ NK cells (6), exhibit a shorter telomere length than their Siglec-9 negative counterparts. In the present study, telomere length analysis of sorted T cell subsets by automated multicolor flow-FISH (15, 26), revealed that the telomere length of Siglec-7^+^ CD8^+^ T cells is around two kilobases shorter than in Siglec-7/9^-/-^ T cells (**Figure 4A**), but similar to the Siglec-9^+^ T cell subset (15).

When redirected against αCD3-coated P815 cancer cells, Siglec-7^+^ CD8^+^ T cells demonstrated a higher cytotoxic capacity than their Siglec-7 negative counterparts (**Figure 4B**), and co-stimulation with αCD3 and αCD28 mAbs resulted in stronger IFN-γ and TNF-α production in Siglec-7^+^ CD8^+^ T cells (**Figures 4C, D**). Flow cytometric analysis revealed broad surface expression of chemokine receptors on Siglec-7^+^ CD8^+^ T cells (**Figure 4E**). Given the significant expression of CCR5 and CXCR3 on this subset, migration towards the chemokines RANTES (CCR5 ligand) and CXCL9 (CXCR3 ligand), was assessed. Indeed, Siglec-7^+^ CD8^+^ T cells demonstrated a higher migration capacity towards these chemokines (**Figures 4F, G**). Together, these data confirm a history of previous clonal expansion for Siglec-7^+^ CD8^+^ T cells and indicate that this subset of effector memory T cells is not exhausted but exhibits high functional and chemotactic capabilities.

### Inhibition of Siglec-7^+^ CD8^+^ T cells by sialoglycans on tumor cells

**Figure 5:**
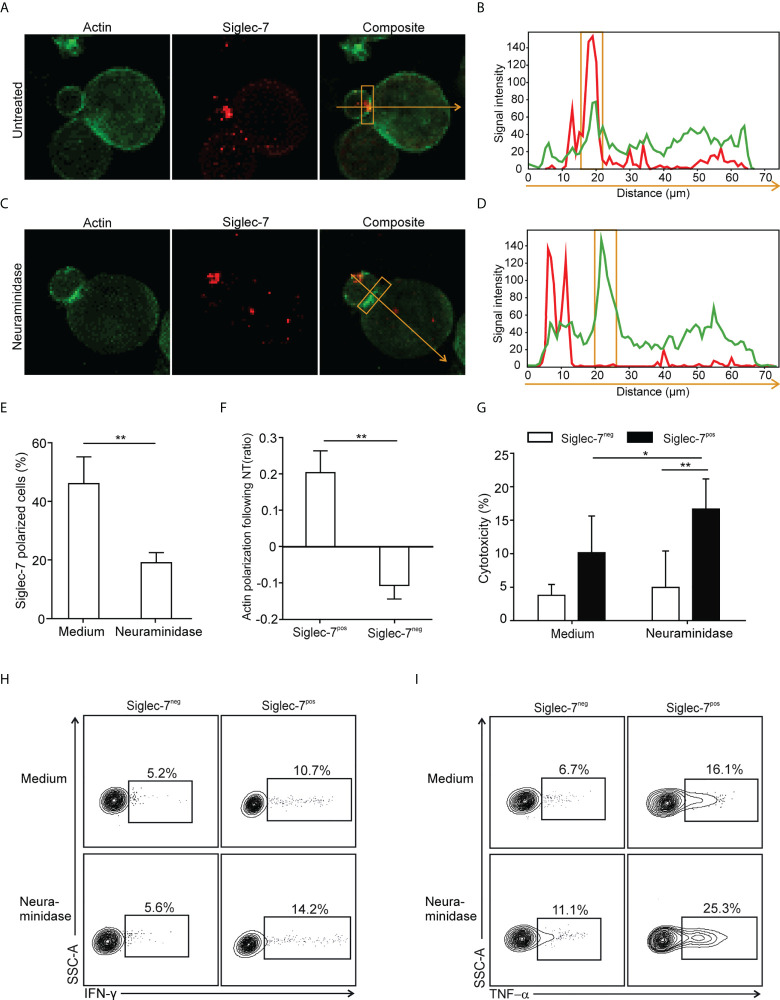
Polarization of Siglec-7 and inhibition of Siglec-7^+^ CD8+ T cells induced by sialoglycans on tumor cells. **(A–F)** Confocal immunofluorescence microscopy analysis of immunological synapse (IS) formation between redirected CD8+ T cell subsets and αCD3 mAb-loaded P815 tumor cells in absence or presence of target cell neuraminidase treatment (NT). Representative images **(A, C)** and corresponding fluorescence profiles plotted along the indicated trajectory **(B, D)**. Quantification of Siglec-7 polarization on T cells towards the IS **(E)**, or actin staining intensity at the synapse **(F)**, compared with the opposite side of the same T cell. **(G–I)** Effects of neuraminidase pre-treatment of P815 target cells on redirected cytotoxicity **(G)** and intracellular IFN-γ **(H)** or TNF−α **(I)** production 4 h after target cell loading with αCD3 mAb (n=5). **(A–F)** analysis of at least 40 conjugates from three independent experiments with cells from at least three donors. Statistical analyses were performed by paired *t* test **(E–G)** or one-way ANOVA followed by Bonferroni posttest **(H, I)**. *P < 0.05; **P < 0.01. Error bars, SD.

The formation of an immune synapse (IS) involves the spatio-temporal organization of molecular events at the interface between effector and target cells. For IS formation analysis by confocal microscopy, sorted CD8^+^ T cell subsets were redirected to αCD3-coated P815 target cells, which express Siglec-7 surface ligands that can be removed by enzymatic digestion with neuraminidase (**Supplementary Figure 5**). We observed polarization of the Siglec-7 receptor into the IS, which was diminished upon neuraminidase treatment (**Figures 5A-E**), indicating the requirement of sialic acid-dependent receptor-ligand interactions *in trans* for effective Siglec-7 polarization. Moreover, neuraminidase treatment of target cells further enhanced actin polarization, required for the establishment of a stable and functional IS (27), in synapses formed with Siglec-7^+^ CD8^+^ T cells but not with Siglec-7^-^ CD8^+^ T cells (**Figure 5F**). Functional experiments revealed that the digestion of Siglec-7 ligands by neuraminidase treatment on αCD3-coated P815 target cells increased effector functions of Siglec-7^+^, but not Siglec-7^-^ CD8^+^ T cells, including redirected cytotoxicity (**Figure 5G**), as well as IFN-γ and TNF-α (**Figures 5H, I**) production. Together, these mechanistic studies provide functional evidence for the sialic acid-Siglec axis as an immune checkpoint that directly regulates effector functions of Siglec-7^+^ CD8^+^ T effector memory cells.

### Siglec-7^+^ T cell glyco-immune checkpoints on AML and leukemic stem cells

**Figure 6:**
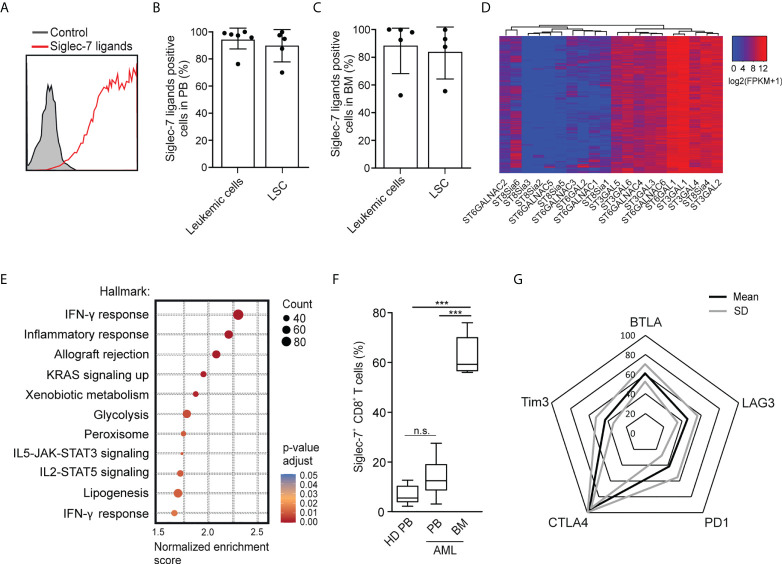
Occurrence of Siglec-7^+^ CD8+ T cells glyco-immune checkpoints in AML. **(A)** Representative flow cytometric histogram demonstrating Siglec-7 ligands surface expression on AML cells isolated from bone marrow (BM). **(B–C)** Siglec-7 ligands expression on AML patient peripheral blood leukemic cells (n=6) and leukemic stem cells (LSC; n=5) **(B)**, or bone marrow leukemic cells (n=5) and leukemic stem cell (LSC; n=4) **(C)**. **(D)** RNA expression of sialyltransferases in AML patients based on TCGA Network data computed by a dendrogram clustering algorithm and **(E)** gene set enrichment analysis performed using hallmark gene sets (n=818). **(F)** Quantitative analysis of Siglec-7 expression by CD8+ T cells from the PB (PB, n=8) and from the bone marrow (BM, n=5) of patients with acute myeloid leukemia (AML). **(G)** Radar chart of flow-cytometric data demonstrating surface coexpression of Siglec-7 with PD1, CTLA4, BTLA, LAG3, or TIM3 on CD8+ T cells from AML bone marrow (n=4). Statistical analyses were performed by one-way ANOVA followed by Bonferroni posttest **(F)** ***P < 0.001; n.s., not significant. Error bars, SD.

Using flow cytometry, we went on to analyze the expression of Siglec-7 ligands on leukemic cells (Lin^-^CD90^-^CD34^+^CD38^+^) and leukemic stem cells (Lin^-^CD90^-^CD34^+^CD38^-^) in AML patient-derived peripheral blood (PB) and bone marrow (BM) (17). High surface expression of Siglec-7 ligands was detected on both AML cells and leukemic stem cells in peripheral blood and bone marrow (**Figures 6A–C**). An analysis of RNA-seq data from the AML data set (n=818) from The Cancer Genome Atlas (TCGA) project using a clustering algorithm revealed heterogenous expression of the twenty human sialyltransferases (**Figure 6D**), which are involved in the biosynthesis of sialoglycans. Consistently high expression levels were found for sialyltransferases (ST) predicted to be involved in the biosynthesis of Siglec-7 ligands, including *ST3GAL1*, *ST3GAL4*, *ST6GAL1*, and *ST6GALNAC6* (27–31). A gene set enrichment analysis of the AML TCGA data revealed a strong correlation between Siglec-7 expression and hallmark gene sets linked to effector CD8^+^ T cell activity, such as IFN-γ response, inflammatory response, glycolysis or IL-2-STAT5 signaling (**Figure 6E**).

Next, we used multi-parametric flow cytometry to explore the expression of Siglec-7 on CD8^+^ T cells in peripheral blood (n=8) and bone marrow (n=5) from AML patients. We observed that the majority of CD8^+^ T cells in the bone marrow of AML patients expressed Siglec-7, while circulating Siglec-7^+^ CD8^+^ T cells in AML patients were found at similar levels as compared to healthy donors (**Figure 6F**). Further analysis of Siglec-7^+^ CD8^+^ T cells isolated from the bone marrow of four AML patients revealed co-expression with CTLA4, and, to a lesser extent with other check-point receptors, such as PD1, LAG3, BTLA, TIM3 (**Figure 6G**). These data suggest a potential role of the sialic acid-Siglec axis as an immune checkpoint in AML.

## Discussion

Increased expression of multiple inhibitory receptors is commonly considered a hallmark of exhausted CD8^+^ T cells (32). However, in this study we observed that in humans Siglec-7 defines a non-exhausted effector memory CD8^+^ T cell subset characterized by high functional and metabolic capacities. For effective functionality, effector memory CD8^+^ T cells need to reprogram their metabolism, which also allows to overcome barriers imposed by challenging environments such as the tumor microenvironment (TME) (33). While exhausted T cells are known to progressively undergo metabolic dysfunction (32), Siglec-7^+^ CD8^+^ T cells were found to exhibit a high potential for concomitant glycolysis and oxidative phosphorylation upon activation, as assessed by ECAR and OCR measurements, respectively. Indeed, Siglec-7^+^ CD8^+^ T cells demonstrated superior functionality in terms of cytotoxicity, cytokine production, and migratory potential compared to Siglec-7 negative cells.

Rather than exhaustion, Siglec-7 expression on CD8^+^ T cells was linked to clonality. Indeed, Siglec-7^+^ CD8^+^ T cells exhibit short telomere length and high TCRvβ chain clonality, which together is indicative of a history extensive of previous clonal expansion. The clonotype repertoire of Siglec-7^+^ CD8^+^ T cells was distinct and showed higher clonality compared to other CD8^+^ T cell subsets, including Siglec-9 positive oligoclonal counterparts found at lower frequency in peripheral blood. Notably, it has been reported that pre-treatment clonality is predictive of response to anti-PD1 treatment, while TCR diversity in tumor-infiltrating lymphocytes (TILs) is prognostic for overall survival in absence of immune checkpoint therapy (34). In line with this observation, results from our study support the conceptual perspective that the expression of at least certain inhibitory receptors is not a defining phenotype of exhausted T cells but is associated with clonality, as a consequence of previous T cell expansion. Furthermore, our data suggest that in combination with T cell repertoire analysis (35), the exploration of clonality-associated immune checkpoints may provide novel avenues for personalized immunotherapy.

Recent advances in immune checkpoint therapy highlight the importance of CD8^+^ T cells in anti-tumor responses and the need for “immune normalization” to restore tumor-induced immune deficiency (34). Current immune checkpoint therapy is successfully used in patient subsets of select solid tumors and lymphomas, but ineffective in other malignancies such as AML in which epigenetic silencing of activating immune checkpoint receptors rather than inhibitory signaling might lead to T cell dysfunction (36). However, in this study we observed high expression of Siglec-7 ligands on leukemic cells and leukemic stem cells in peripheral blood and bone marrow from AML patients. Intriguingly, the majority of CD8^+^ T cells in the bone marrow of AML patients expressed Siglec-7, with high co-expression of CTLA4, but lower expression of other inhibitory receptors (PD1, LAG3, BTLA, TIM3). These findings suggest that the sialic acid-Siglec axis provides a glyco-immune checkpoint in AML that restrains effective anti-tumor responses of CD8+ T cells in the bone marrow environment. Indeed, in mechanistic experiments, we observed Siglec-7 repolarization on CD8+ T cells into the immune synapse with target cells expressing the cognate ligand, as well as sialic-acid dependent suppression of cytotoxicity and cytokine production selectively of Siglec-7^+^ CD8+ T cells.

There is increasing awareness that reverse translational research from bedside to bench is needed for more personalized pharmacotherapy and to explore the complexity of processes at play in human diseases (37). In this study, we identified Siglec-7 as an immune checkpoint receptor on non-exhausted effector memory CD8+ T cells that is acquired upon clonal expansion. Strategies considering the combined analysis of T cell repertoires and clonality-associated immune checkpoint receptors, such as Siglec-7, may lead to novel and more personalized treatment approaches for T cell-driven autoimmune disorders and for cancer immunotherapy.

## Data availability statement

The data presented in the study are deposited in the immuneACCESS repository, accession number haas-2022-fi (DOI:10.21417; URL: https://clients.adaptivebiotech.com/pub/haas-2022-fi).

## Ethics statement

The studies involving human participants were reviewed and approved by the ethics committee of the canton of Bern, Switzerland. The patients/participants provided their written informed consent to participate in this study.

## Author contributions

SG designed the study. QH and SG wrote the manuscript. TCGA data were analyzed by AB, CN and QH. Experimental work was performed by QH, NM, LM, MH, and VR under supervision by SG, H-US, GB, CR and CM. Patient material was provided by CR and AO. All authors had full access to the data, helped draft the report or critically revised the draft, contributed to data interpretation, reviewed and approved the final version of the report.

## Funding

The laboratory of SG was supported by grants from the Swiss National Science Foundation (SNSF) [310030_184757], Swiss Cancer League/Swiss Cancer Research [KFS-4958-02-2020], Palleon Pharmaceuticals Inc., Waltham MA (USA), Mizutani Foundation for Glycoscience, the Novartis Research Foundation and the Bern Center for Precision Medicine (BCPM). HU-S received support from the Swiss National Science Foundation (grant No. 310030-166473 and 310030_184816), the European Union’s Horizon 2020 research and innovation program (Marie Sklodowska-Curie grant No. 642295; MEL-PLEX) and the Russian Government Program “Recruitment of the Leading Scientists into the Russian Institutions of Higher Education”, grant No. 075-15-2021-600 (HUS). CM receives financial support from Cancer Research Switzerland (KFS-4962-02-2020), HMZ ImmunoTargET of the University of Zurich, the Cancer Research Center Zurich, the Sobek Foundation, the Swiss Vaccine Research Institute, the Swiss MS Society (2021-09), Roche, Novartis, Innosuisse (52533.1), and the Swiss National Science Foundation (310030_204470/1, 310030L_197952/1 and CRSII5_180323). Palleon Pharmaceuticals and Novartis were not involved in study design, collection, analysis, interpretation of data, the writing of this article, or decision to submit it for publication.

## Acknowledgments

We thank Ingrid Helsen for technical assistance with the telomere length analysis.

## Conflict of interest

SG receives remuneration for serving on the scientific advisory board of Palleon Pharmaceuticals.

The remaining authors declare that the research was conducted in the absence of any commercial or financial relationships that could be construed as a potential conflict of interest.

## Publisher’s note

All claims expressed in this article are solely those of the authors and do not necessarily represent those of their affiliated organizations, or those of the publisher, the editors and the reviewers. Any product that may be evaluated in this article, or claim that may be made by its manufacturer, is not guaranteed or endorsed by the publisher.
