# Supplementary material for: Siglec-7 represents a glyco-immune checkpoint for non-exhausted effector memory CD8+ T cells with high functional and metabolic capacities
Source: Front Immunol. 2022 Sep 23;13:996746. doi: 10.3389/fimmu.2022.996746 (PMC9540514; doi:10.3389/fimmu.2022.996746)
Supplement: Supplementary file 1 [file DataSheet_1.pdf]

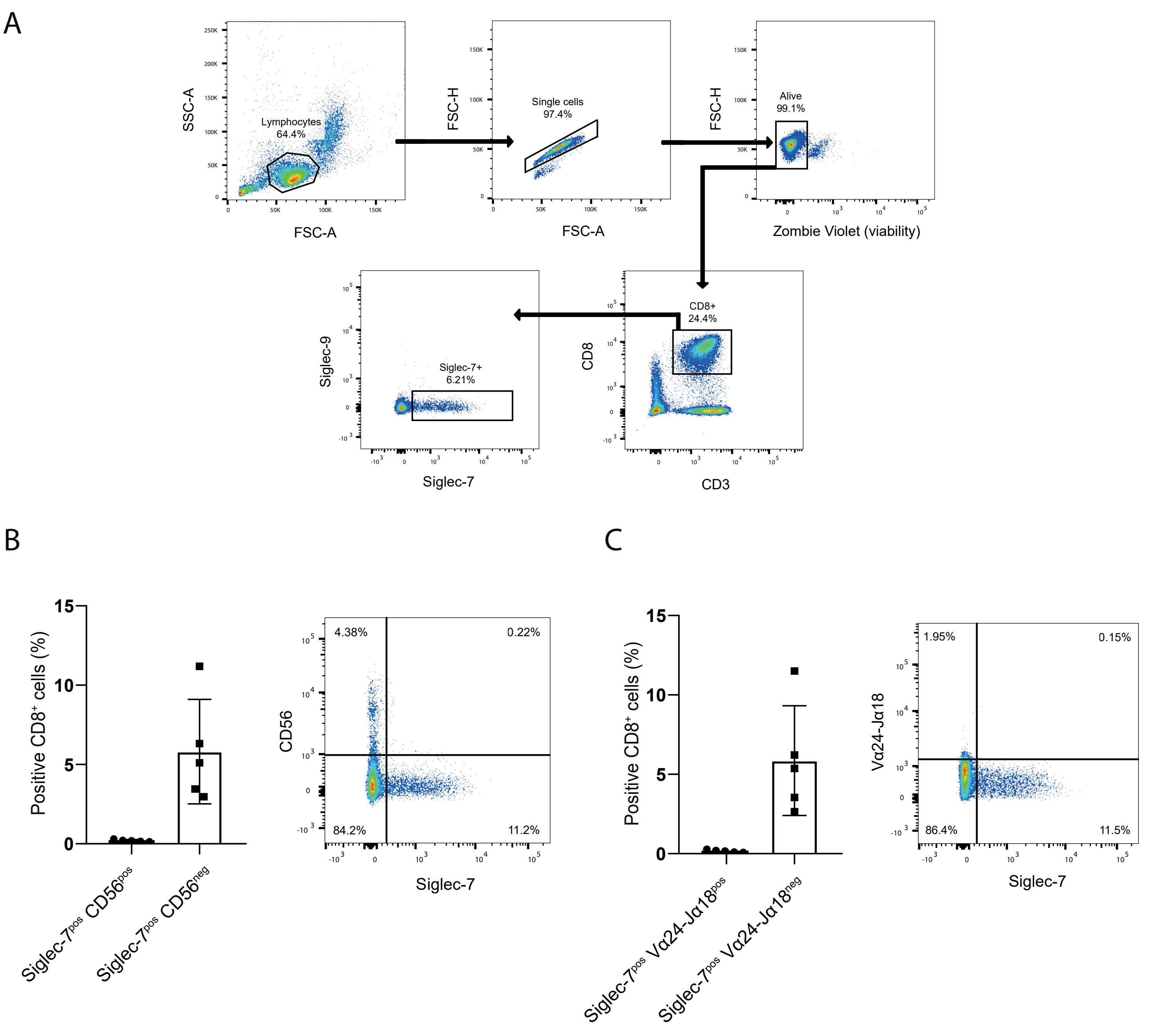

Supplementary figure 1. (A) Gating strategy for flow cytometric analysis. (B, C) Surface co-staining of Siglec-7 and CD56 (B) or TCR Vα24-Jα18 (C) on peripheral blood $CD8^+$ T cells from healthy donors (n=5), as assessed by flow cytometry.

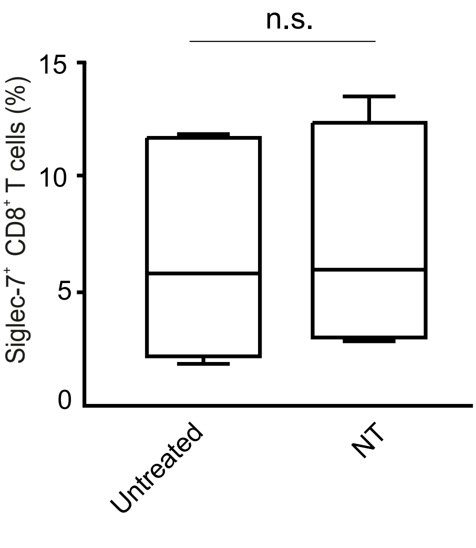

Supplementary figure 2. Quantitative flow cytomertric analysis of Siglec-7 surface expression on peripheral blood CD8+ T cells from healthy donors (n=5) with or without neuraminidase treatment (NT). Student *t*-test, n.s., not significant.

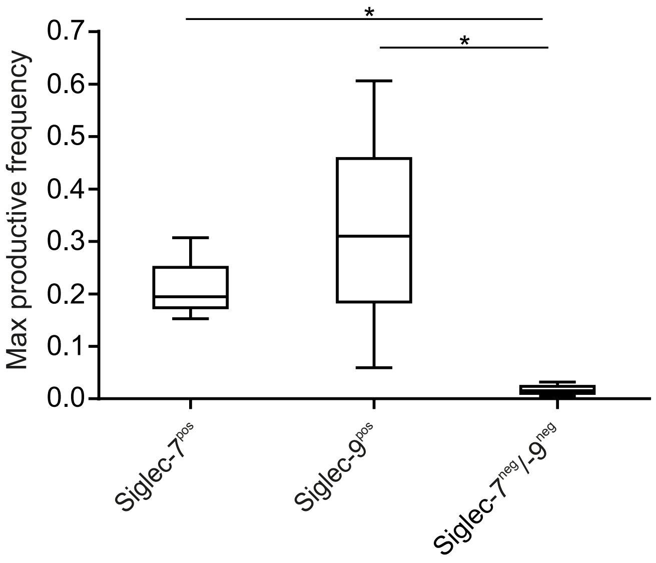

Supplementary figure 3. Maximum productive frequency of clonotypes among Siglec-7^{+}, Siglec-9^{+},and Siglec-7/9$^{-/-}$ CD8^{+} T cells isolated from healthy donors peripheral blood (n=3). One-way ANOVA followed by Bonferroni posttest. *, $P < 0.05$.

A

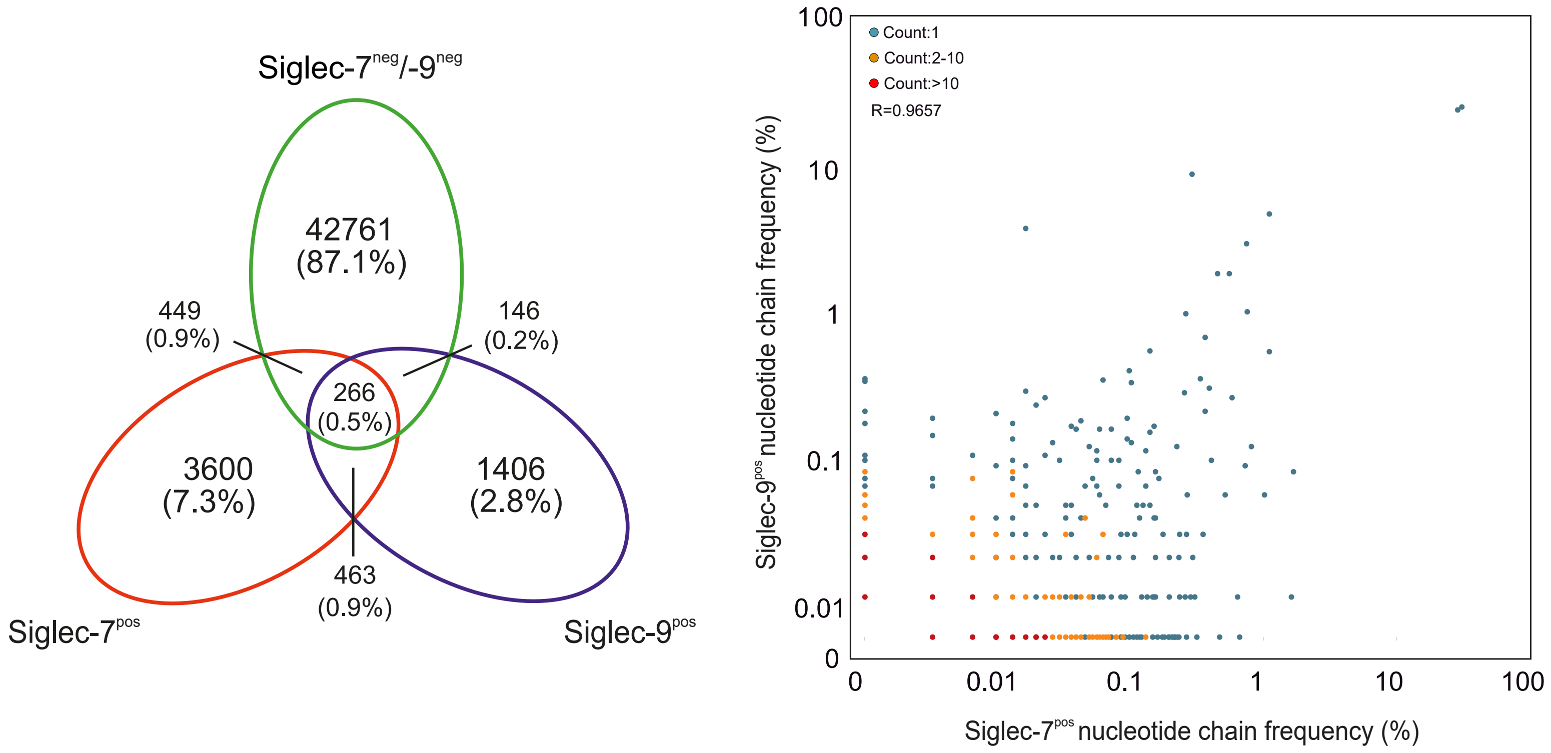

B

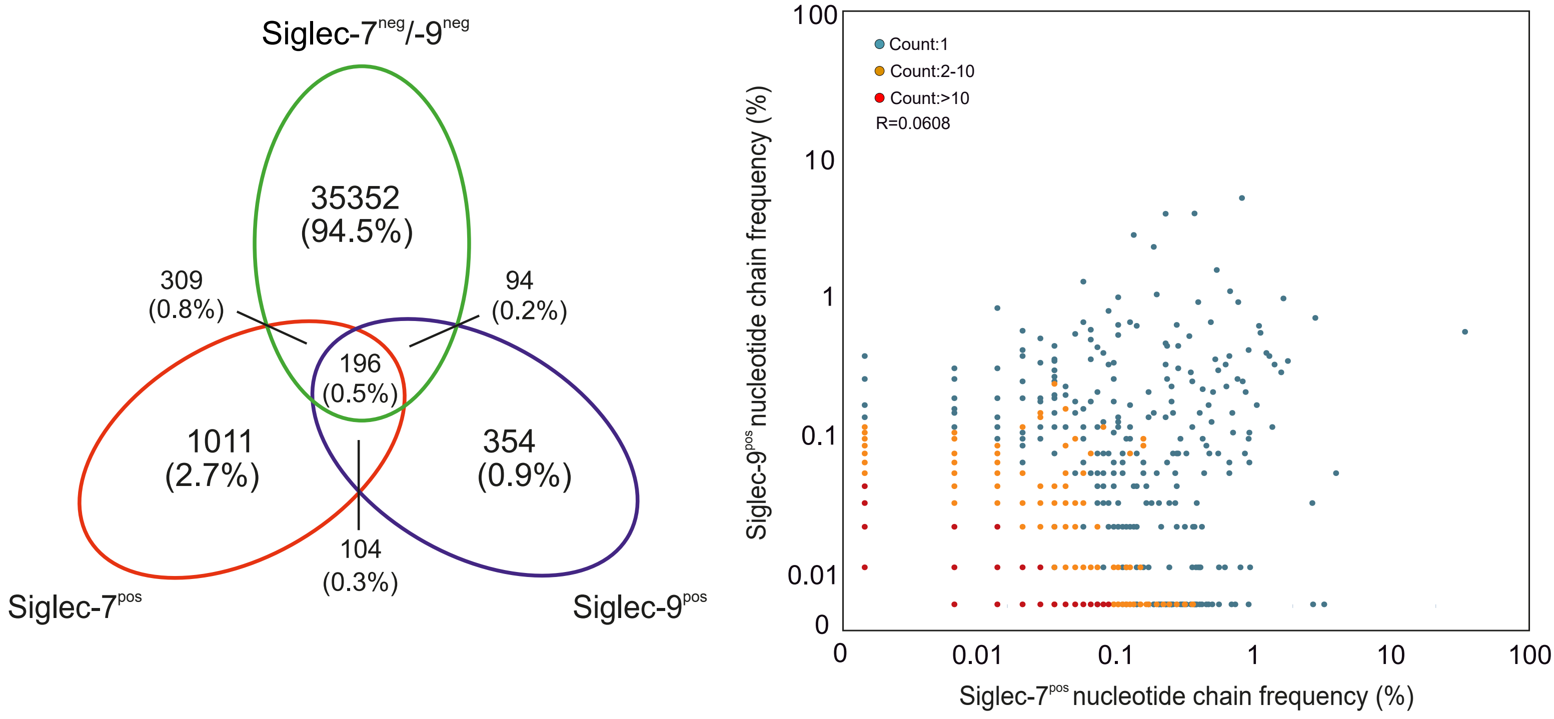

Supplementary figure 4. Venn diagram and scatter plot representation displaying clonotype distribution among $CD8^+$ T cell subsets for donor TS-01 (A) and TS-02 (B).

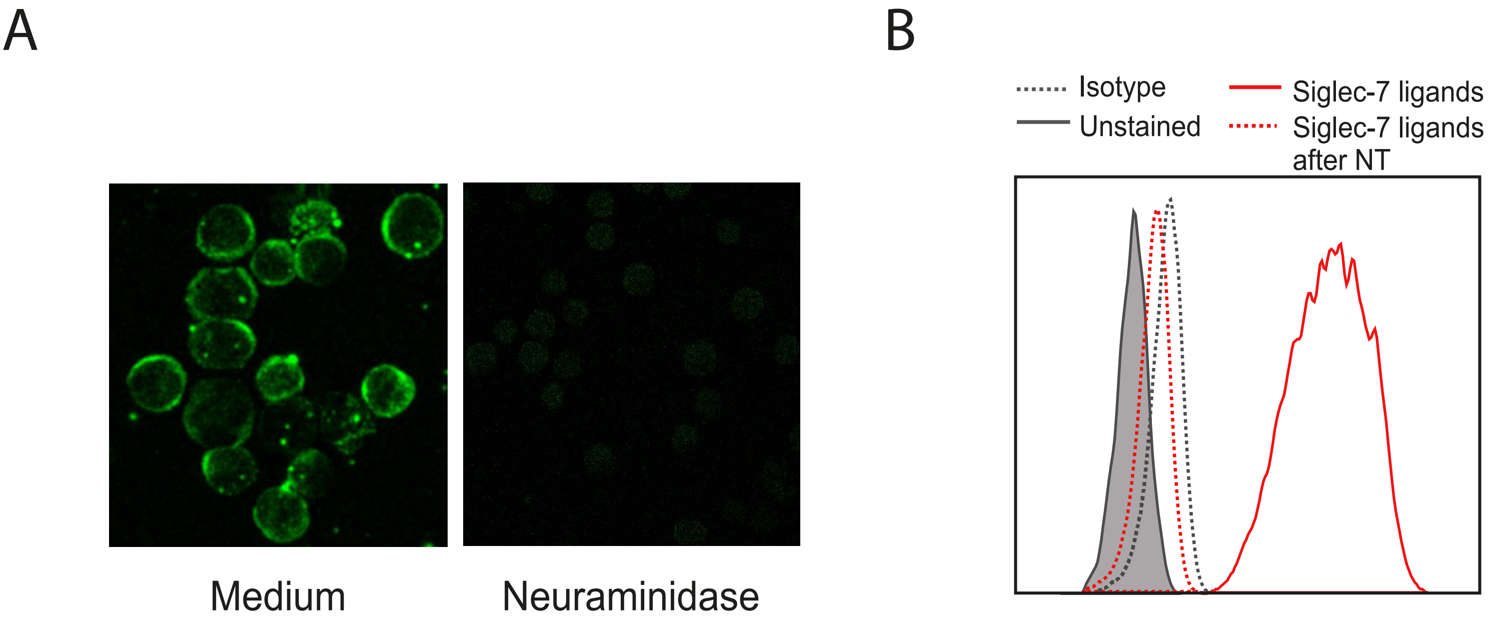

Supplementary figure 5. Confocal microscopy (A) and flow cytometric (B) analysis of Siglec-7 ligands expression on P815 cells with and without neuraminidase treatment (NT).
